# Reproducibility of 2D versus 3D radiomics for quantitative assessment of fetal lung development: a retrospective fetal MRI study

**DOI:** 10.1186/s13244-023-01376-y

**Published:** 2023-02-08

**Authors:** Martin L. Watzenboeck, Benedikt H. Heidinger, Julian Rainer, Victor Schmidbauer, Barbara Ulm, Erika Rubesova, Daniela Prayer, Gregor Kasprian, Florian Prayer

**Affiliations:** 1 Department of Biomedical Imaging and Image-Guided Therapy, Medical University of Vienna, Währinger Gürtel 18-20, 1090 Vienna, Austria; 2 Department of Obstetrics and Gynecology, Medical University of Vienna, Spitalgasse 23, Währinger Gürtel 18-20, 1090 Vienna, Austria; 3 Department of Pediatric Radiology, Lucile Packard Children’s Hospital at Stanford, Stanford University, 725 Welch Road, Stanford, CA 94305 USA; 4 Imaging Bellaria, Bellariastrasse 3, 1010 Vienna, Austria

**Keywords:** Magnetic resonance imaging, Fetal development, Lung, Reproducibility of results, Radiomics

## Abstract

**Purpose:**

To investigate the reproducibility of radiomics features extracted from two-dimensional regions of interest (2D ROIs) versus whole lung (3D) ROIs in repeated in-vivo fetal magnetic resonance imaging (MRI) acquisitions.

**Methods:**

Thirty fetal MRI scans including two axial T2-weighted acquisitions of the lungs were analysed. 2D (lung at the level of the carina) and 3D (whole lung) ROIs were manually segmented using ITK-Snap. Ninety-five radiomics features were extracted from 2 and 3D ROIs in initial and repeat acquisitions using Pyradiomics. Radiomics feature intra-class correlation coefficients (ICC) were calculated between 2 and 3D ROIs in the initial acquisition, and between 2 and 3D ROIs in repeated acquisitions, respectively.

**Results:**

MRI data of 11 (36.7%) female and 19 (63.3%) male fetuses acquired at a median 25 + 0 gestational weeks plus days (GW) (interquartile range [IQR] 23 + 4 − 27 + 0 GW) were assessed. Median radiomics feature ICC between 2 and 3D ROIs in the initial MRI acquisition was 0.733 (IQR 0.313–0.814, range 0.018–0.970). ICCs between radiomics features extracted using 3D ROIs in initial and repeat acquisitions (median 0.908 [IQR 0.824–0.929, range 0.335–0.996]) were significantly higher compared to 2D ROIs (0.771 [0.699–0.835, 0.048–0.965]) (*p* < 0.001).

**Conclusion:**

Fetal MRI radiomics features extracted from 3D whole lung segmentation masks showed significantly higher reproducibility across repeat acquisitions compared to 2D ROIs. Therefore, fetal MRI whole lung radiomics features are robust diagnostic and potentially prognostic tools in the image-based in-vivo quantitative assessment of lung development.

**Supplementary Information:**

The online version contains supplementary material available at 10.1186/s13244-023-01376-y.

## Key points

- Standardised fetal MRI delivers reproducible quantitative whole lung radiomics features.
- 2D lung ROI radiomics features are not representative of the whole fetal lung.
- Whole lung radiomics features are better reproducible than 2D ROI features.
- Whole lung radiomics features are more sensitive to subtle differences between normal and pathological fetal lung development compared to 2D lung ROI features.
- Fetal MRI whole lung radiomics may improve non-invasive in-vivo lung development assessment.

**Supplementary Information:**

The online version contains supplementary material available at 10.1186/s13244-023-01376-y.

## Background

Prenatal detection of pathologic lung development is a prerequisite for timely resource allocation, including delivery in a centre offering advanced neonatal respiratory support techniques such as extra-corporeal membrane oxygenation. Currently, non-invasive in-vivo imaging assessment of fetal lung growth relies primarily on fetal ultrasound or magnetic resonance imaging (MRI) based lung volumetry [1]. This approach is limited by wide gestational age and body volume-adjusted normal lung volume ranges that may only inaccurately identify fetuses at risk for postnatal respiratory insufficiency [2, 3]. Research on fetal MRI signal intensity-based analysis of lung maturity has produced variable results, thus far precluding its clinical translation [4].

The use of radiomics, a quantitative image analysis method that is used to extract a large number of features from medical image data, has been proposed to increase the diagnostic and prognostic imaging yield in a variety of conditions [5–7]. Recently, studies have highlighted the potential of radiomics for the identification of fetuses with abnormal lung development and texture-based neonatal respiratory distress prediction [8–10]. Thus far, fetal lung radiomics features have been extracted from two-dimensional (2D), representative-appearing regions of interest (ROI) in fetal lung ultrasound images [10]. However, it is unclear if features extracted from 2D ROIs are sufficient to reflect the developmental status of the entire fetal lung. In addition, reproducibility of radiomics features in test–retest conditions is a precondition for their safe and meaningful application according to the Radiomics Quality Score proposed by Lambin et al. [11]. To date, there is a lack of evidence regarding the reproducibility of radiomics features extracted from the fetal lung using 2D ROIs, limiting the wider application of radiomics in fetal lung imaging and beyond. Fetal MRI offers the opportunity to obtain three-dimensional (3D) image data of the developing lungs in a standardised fashion, and may facilitate reproducible radiomics-based quantitative assessment of lung development.

Therefore, this study had two aims: First, to investigate whether fetal MRI lung radiomics features extracted from 2D ROIs are representative of features extracted from whole lung segmentation masks (3D ROIs). Second, to investigate the reproducibility of lung radiomics features extracted from 2 and 3D ROIs in repeated standardised fetal MRI acquisitions.

## Methods

This retrospective, single-centre study was approved by the Ethics Committee of the Medical University of Vienna (1232/2022). The requirement to obtain informed consent was waived. A part of the study cohort (*n* = 29) has been previously reported in a study that did not include 2D radiomics feature analyses [12].

### Patients

A retrospective search of the hospital picture and archiving system (PACS) was conducted to identify thirty cases that underwent clinically indicated fetal MRI including two axial T2-weighted acquisitions of the developing lung between January of 2016 and February of 2022. The sample size was chosen in accordance with previous test–retest studies investigating radiomics feature reproducibility [13–15]. In order to assess radiomics feature reproducibility, cases with normal and diffuse lung pathology (e.g. pulmonary hypoplasia due to premature rupture of membranes) at different gestational ages were included. Cases were excluded from analysis for lack of ultrasound-based gestational age estimation, presence of a focal lung malformation, incomplete lung representation on fetal MRI, lung tissue visible on five or fewer MRI slices, or presence of (fetal or maternal) motion artifacts.

### Fetal MRI

Fetal MRI data from clinically indicated scans were retrospectively identified. Repeated standardised axial T2-weighted acquisitions of the lungs were routinely performed for e.g. lung volumetry or data collection for super-resolution body imaging according to the clinical indication and in accordance with ISUOG guidelines [16]. One 1.5 T MRI scanner (Ingenia, Philips Healthcare, Best, The Netherlands) was used for all exams. Axial T2-weighted acquisitions were acquired in a standardised fashion using a body coil and the following parameters: 200 to 300 mm field of view, 3 to 4 mm slice thickness (thinner slices used in early gestation), 0.3 to 0.4 mm gap, 256 × 256 matrix, shortest (7536.2 to 31,575 ms) repetition time, 100 ms echo time, and 90° flip angle. Specific absorption rates were less than 2W/kg for all cases. Fetal MRI scans were performed without administration of sedation or contrast medium. The time in minutes between initial and repeat axial T2-weighted acquisitions was calculated for each case. Gestational age in weeks plus days (GW) post menstruationem at the time of the fetal MRI scan was calculated based on the first fetal ultrasound examination.

### Radiomics

**Fig. 1:**
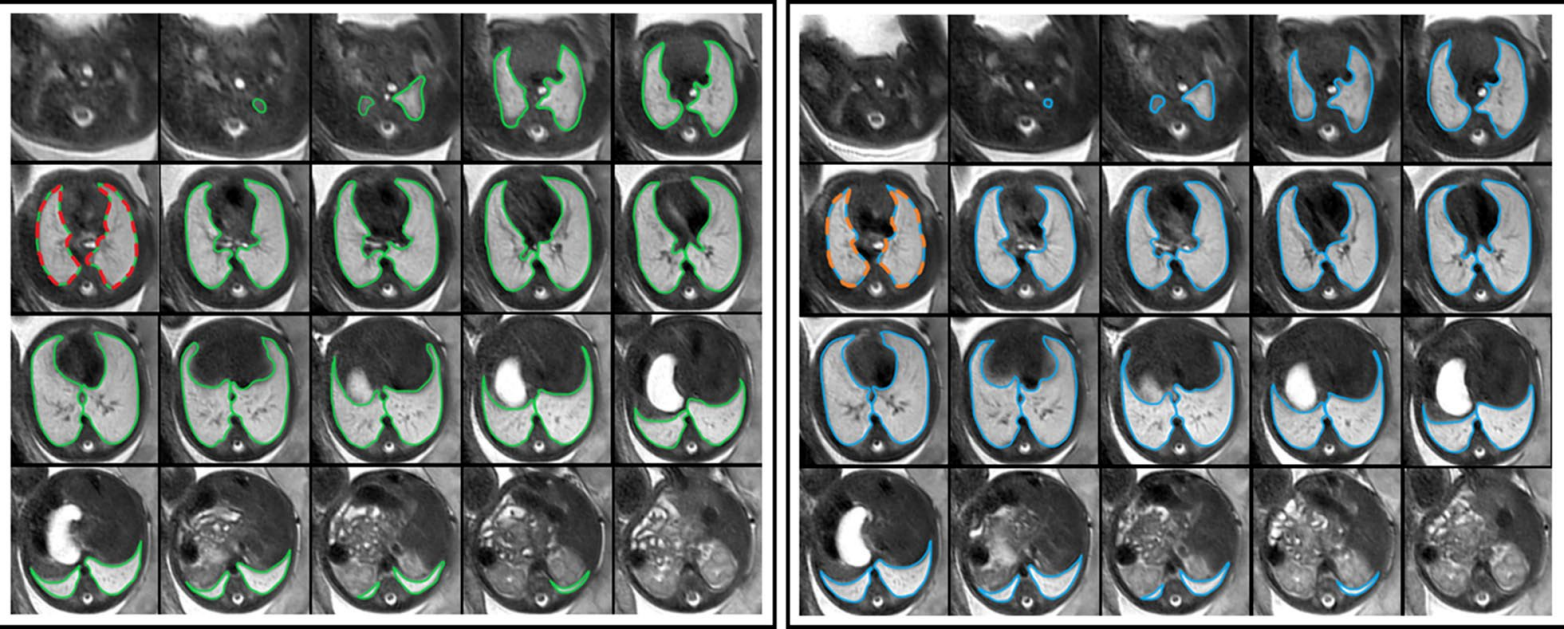
Study design. Repeated acquisitions of T2-weighted axial MRI images of the lung in a fetus at gestational week 32. Three-dimensional (3D) regions of interest (ROIs) encompassing the whole lung were manually segmented in initial (green) and repeat (blue) acquisitions of 30 fetuses. Two-dimensional (2D) ROIs were defined as lung segmentations at the level of the carina in initial (red) and repeat (orange) acquisitions, respectively. Radiomics feature reproducibility was assessed between features extracted from 2 and 3D ROIs in the initial acquisition (red vs. green), 2D ROIs in repeated acquisitions (red vs. orange), and 3D ROIs in repeated acquisitions (green vs. blue)

Anonymised fetal MRI data were exported from the hospital PACS (Dedalus HealthCare, Bonn, Germany). Manual segmentation masks of the whole lung (3D ROI) were obtained for initial and repeat T2-weighted axial fetal MRI acquisitions by one radiologist with five years of experience in fetal MRI (F.P.) using ITK-Snap [17]. For each 3D ROI, the slice index at the level of the carina was recorded. MRI images and lung segmentation masks were saved as nifti-files. In addition, image and lung segmentation mask slices at the level of the carina were converted to 2D nifti-files using the python package nibabel (MIT). Radiomics features were extracted from 2 and 3D ROIs using Pyradiomics [18], under Python 3.7.1 with the following settings: normalise parameter 'true', normalise Scale parameter 100, voxelArrayShift 300, (3 SDs × 100) ensuring that only outlier values > 3 SDs below the mean remain negative, binWidth 5, 'sitkBSpline' as interpolator, and resampledPixelSpacing '[2, 2, 2]' for 3D or ‘[2, 2]’ for 2D image data. Radiomics features encompassed the following classes: First Order (*n* = 18), 3D or 2D Shape (*n* = 9), Grey Level Co-occurrence Matrix (GLCM, *n* = 22), Grey Level Size Zone Matrix (GLSZM, *n* = 16), Grey Level Run Length Matrix (GLRLM, *n* = 16), and Grey Level Dependence Matrix (GLDM, *n* = 14). A list of all radiomics features is provided in the Additional file 1. See Fig. 1 for an illustration of the study design.

### Statistical evaluation

R version 4.0.5 (R Core Team, Vienna, Austria) was used for statistical analysis. Intra-class correlation coefficients (ICC) were calculated to assess radiomics feature reproducibility within the initial MRI acquisition between 2 and 3D ROIs, and between initial and repeat MRI acquisition for 2D vs 2D ROIs, and 3D versus 3D ROIs, respectively. The psych R package (version 2.1.9) was used to calculate ICCs, applying two-way mixed effects models (ICC3) and single rater unit. ICCs > 0.9 were considered excellent, > 0.75 to 0.9 good, 0.5 to 0.75 moderate, and < 0.5 poor according to Koo et al. [19]. The paired Wilcoxon Rank Sum test was used to compare ICCs of radiomics feature extracted from 2D ROIs in initial and repeat MRI acquisitions, and of radiomics features extracted from 3D ROIs in initial and repeat MRI acquisitions. Differences in radiomics features between fetuses with pathological or healthy lung development were assessed using ANCOVA followed by estimated marginal mean (EMM, emmeans R package) comparisons, whereby gestational age was included as a covariate and binned into 20 day intervals (< 160, 160–180, 180–200, 200–220 and > 220 days) to control for potential non-linear growth effects. EMM comparison *p* values were corrected for multiple testing using the false discovery rate (FDR). A two-sided *p* value of < 0.05 or a false discovery rate of < 0.1 combined with a two-sided *p* value of < 0.05, as applicable, were considered statistically significant.

## Results

**Fig. 2:**
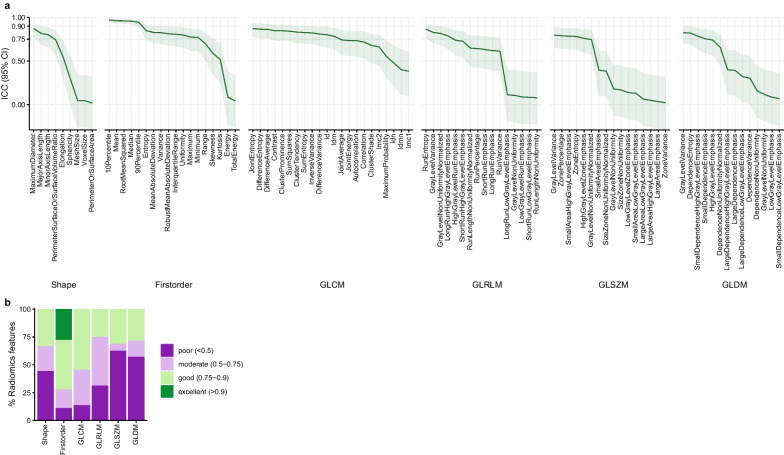
2D versus 3D fetal MRI lung radiomics features. **a** Intra-class correlation coefficients (ICCs) and 95% confidence intervals (CI) between 2 and 3D ROIs in a single MRI acquisition for each of the 95 investigated radiomics features. Lines indicate ICCs, and ribbons 95% CI. Features are grouped according to class. **b** Barplots depicting the proportions of radiomics features with poor (ICC < 0.5), moderate (0.5–0.75), good (0.75–0.9) and excellent (> 0.9) reproducibility grouped according to class; CI confidence interval GLCM: grey level co-occurrence matrix, GLDM: grey level dependence matrix, GLRLM: grey level run length matrix, GLSZM: grey level size zone matrix, ICC intra-class correlation coefficient

Key characteristics of the study cohort are given in Table 1. The median time between initial and repeat axial T2-weighted MRI acquisitions was 3.7 min (IQR 0.9–5.9 min, range 0.7–24.2 min). Radiomics features extracted from 2 and 3D ROIs in the initial MRI acquisition were reproducible to a variable degree (Fig. 2A, B): The median ICC across all features was 0.733 (IQR 0.313–0.814, range 0.018–0.970). Five of 95 (5.3%) features showed excellent, 36 (37.9%) good, 22 (23.2%) moderate, and 32 (33.7%) poor reproducibility. All five features with excellent reproducibility belonged to the class ‘First Order’ (Fig. 2B).

**Table 1:** Study cohort characteristics

Sex	Female	11/30 (36.7%)
	Male	19/30 (63.3%)
Age	Fetal	25 + 0 GW (IQR 3 + 3)
	Maternal	29.7 (IQR 7.3)
Normal lung development*		22/30 (73.3%)
	Normal fetal development	9/30 (30%)
	Ventricular asymmetry	3/30 (10%)
	Ventriculomegaly	1/30 (3.3%)
	Macrocephaly	1/30 (3.3%)
	Diastematomyelia	1/30 (3.3%)
	Cleft lip and palate	1/30 (3.3%)
	Cardiomegaly	1/30 (3.3%)
	Hypoplastic right heart	1/30 (3.3%)
	Transposition of great arteries	1/30 (3.3%)
	Unilateral ureteral stenosis	1/30 (3.3%)
	Posterior ureteral valve	1/30 (3.3%)
	Muscular atrophy	1/30 (3.3%)
Pathologic lung development		8/30 (26.7%)
	Pulmonary hypoplasia due to premature rupture of membranes and oligohydramnios	6/30 (20%)
	Pulmonary hypoplasia due to hypoplastic kidneys and oligohydramnios	1/30 (3.3%)
	Pulmonary hypoplasia due to autosomal recessive polycystic kidney disease and oligohydramnios	1/30 (3.3%)

Key characteristics of the study cohort: Fetal and maternal ages are given as median and interquartile range

*GW* gestational week plus days post menstruationem, *IQR* interquartile range

*Fetal lung development was considered normal if lung volume was within gestational age-adjusted normal volume ranges

**Fig. 3:**
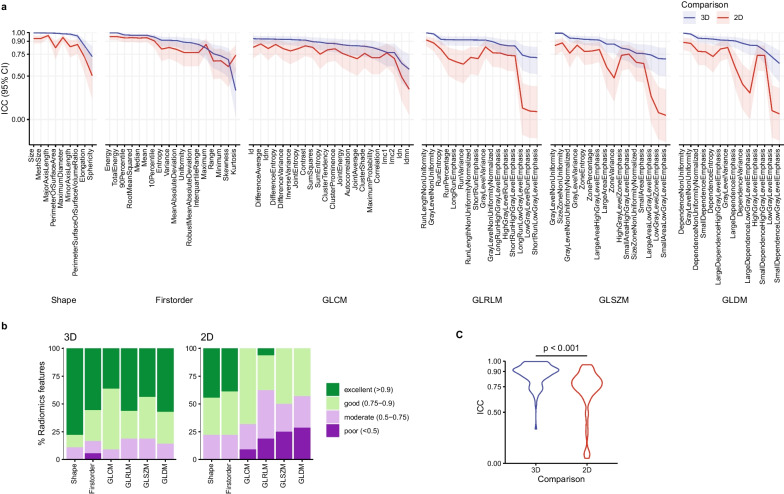
Reproducibility of 2D and 3D fetal MRI lung radiomics features. **a** Intra-class correlation coefficients (ICCs) and 95% confidence intervals (CI) between 2 and 3D regions of interest (ROIs) in repeat examinations for each of the 95 investigated features. Lines (ICCs) and ribbons (95% CI) are coloured according to the comparisons depicted (3D blue, 2D red). Features are grouped according to class. **b** Barplots depicting the proportions of radiomics features with poor (ICC < 0.5), moderate (0.5–0.75), good (0.75–0.9) and excellent (> 0.9) reproducibility according to feature class and 2D or 3D ROIs in repeat examinations. **c** Radiomics feature ICCs were significantly higher between initial and repeat MRI acquisitions if 3D ROIs were used for radiomics feature extraction compared to 2D ROIs; CI confidence interval GLCM: grey level co-occurrence matrix, GLDM: grey level dependence matrix, GLRLM: grey level run length matrix, GLSZM: grey level size zone matrix, ICC intra-class correlation coefficient

Radiomics features extracted from 2D ROIs in initial and repeat MRI acquisitions exhibited a median ICC of 0.771 (IQR 0.699–0.835, range 0.048–0.965; see Fig. 3A). Twelve of 95 (12.6%) features showed excellent, 44 (46.3%) good, 26 (27.4%) moderate, 13 (13.7%) poor reproducibility (see Fig. 3B). Radiomics features extracted from 3D ROIs in initial and repeat MRI acquisitions exhibited a median ICC of 0.908 (IQR 0.824–0.929, range 0.335–0.996). Forty-nine of 95 (51.6%) showed excellent, 32 (33.7%) good, 13 (13.7%) moderate, and 1 (1.1%) poor reproducibility (see Fig. 3B). Table 2 shows radiomics feature reproducibility according to feature classes.

**Table 2:** Radiomics feature reproducibility

MRI acquisitions	First order statistics		Shape features		Texture features	
	ICC > 0.9	ICC ≤ 0.9	ICC > 0.9	ICC ≤ 0.9	ICC > 0.9	ICC ≤ 0.9
*Single*						
2D versus 3D ROI	5/18 (27.8%)	13/18 (72.2%)	0/9 (0%)	9/9 (100%)	0/68 (0%)	68/68 (100%)
*Repeated*						
2D versus 2D ROI	7/18 (38.9%)	11/18 (61.1%)	4/9 (44.4%)	5/9 (55.6%)	1/68 (1.5%)	67/68 (98.5%)
3D versus 3D ROI	10/18 (55.6%)	8/18 (44.4%)	7/9 (77.8%)	2/9 (22.2%)	32/68 (47.1%)	36/68 (52.9%)

Summary of radiomics feature reproducibility for the use of two-and three-dimensional regions of interest in a single and in repeated MRI acquisitions. Excellent reproducibility was defined as intra-class correlation coefficient > 0.9. Texture features include the following classes: Grey Level Co-occurrence Matrix, Grey Level Size Zone Matrix, Grey Level Run Length Matrix, and Grey Level Dependence Matrix

*ICC* intra-class correlation coefficient, *ROI* region of interest

ICCs were significantly higher between initial and repeat MRI acquisitions if 3D ROIs were used for radiomics feature extraction compared to 2D ROIs (*p* < 0.001) (see Fig. 3C). A complete list of radiomics feature ICCs between 2 and 3D ROIs in initial MRI acquisition, and between initial and repeat MRI acquisitions using 2D ROIs, and 3D ROIs is provided in the Additional file 1: Table S1.

Means and standard deviations of radiomics features for all fetuses, and for fetuses with normal and pathological lung development, as well as raw and FDR-corrected *p* values are shown in Additional file 1: Tables S2 (for 3D ROIs) and S3 (for 2D ROIs). Eleven of 100 features (11%) were found to be significantly different between fetuses with normal and pathological lung development when using 3D ROIs (see Additional file 1: Fig. S1). Meanwhile, none of the 100 features (0%) passed the nominal threshold for statistical significance when using 2D ROIs.

## Discussion

Radiomics have the potential to enhance the assessment of fetal MRI data by extracting quantitative image features that may not be visually perceivable [20]. However, reliable radiomics-assisted fetal-MRI-based tissue characterisation requires excellent feature reproducibility [11]. The presented findings show that the use of 2D versus 3D lung ROIs for radiomics feature extraction from fetal MRI data severely impacts feature values. In addition, radiomics features extracted from 3D ROIs encompassing the whole fetal lung outperformed features extracted from 2D lung ROIs with regard to reproducibility in repeated image acquisitions. Therefore, in the future, highly-reproducible fetal MRI radiomics features extracted from whole lung segmentation masks may improve non-invasive quantitative assessment of lung development.

Non-invasive in-vivo MRI assessment of the fetal lungs is safe and feasible during the second and third trimesters, which corresponds to the canalicular and saccular phases of lung development [1]. During this time, besides volume growth, lung organogenesis is characterised by microstructural changes including the formation of pulmonary acini, differentiation of type I and II pneumocytes, and increasing production of lung fluid and surfactant. This can be observed in fetal MRI as an increase in lung volume along with an increase in signal intensity [21]. However, visual assessment of fetal lung signal intensity in MRI is subjective. Integration of quantitative measures of tissue characteristics in the form of lung-to-liver, lung-to-muscle, or lung-to spinal fluid signal intensity ratios into fetal MRI lung assessment have produced mixed results [4, 22–24], so far prohibiting their translation into clinical routine. Therefore, current image-based assessment of fetal lung development focuses primarily on tissue quantity in the form of lung volume rather than tissue quality. Unfortunately, volume alone is an imperfect descriptor of lung developmental status as gestational age-adjusted growth curves show wide normal ranges [2]. Recently, the use of novel fetal MRI techniques including diffusion-weighted imaging [25], intra-voxel incoherent motion analysis [26] and T2* mapping [27] for the microstructural characterisation of fetal lung tissue has been advocated but their benefit remains largely unclear.

In order to facilitate non-invasive, image-based and timely detection of abnormal fetal lung development, reliable quantitative lung tissue features beyond volume are needed. Radiomics allows the extraction of a multitude of features reflecting various aspects of shape and texture from 2D or 3D image ROIs [28]. Fetal MRI is ideally suited for the extraction of quantitative lung radiomics features since image acquisition follows a standardised protocol. Most fetal imaging centres use a single MRI scanner, which has been shown to be essential for radiomics feature reproducibility. Moreover, dedicated fetal MRI lung assessments already requires manual whole lung segmentation to obtain lung volume [29]. These lung segmentations could be integrated into a post-processing pipeline for radiomics feature extraction that has potential for automation due to the high level of standardisation recommended by the Image Biomarker Standardisation Initiative [30]. Therefore, fetal MRI radiomics analysis of lung development could be implemented without the need for additional costly resources.

Fetal lung texture analysis has thus far only been explored using ultrasound images: Previous studies assessed lung maturity [31], identified cases at risk for pulmonary hypoplasia [32], and predicted neonatal respiratory insufficiency [33] with promising results. However, ultrasound studies used 2D fetal lung ROIs to extract texture features, e.g. from lung tissue at the level of the four chamber view. In addition, physicians placed ultrasound ROIs in ‘representative lung areas’ while avoiding artifacts. This approach raises questions with regard to the reliability of radiomics-based fetal lung assessment: Cardiac position (and the level of the four chamber view) may vary in pathologies where lung development is of particular interest, such as in fetuses with congenital diaphragmatic hernia, or congenital pulmonary airway malformation. Furthermore, it is unclear whether a two-dimensional ROI sufficiently represents tissue characteristics of a much larger three-dimensional structure, particularly if subjective criteria are used to determine the ROI’s location. Lastly, there is a lack of evidence regarding the reproducibility of radiomics features extracted from 2D ROIs in repeated image data acquisitions.

The presented results suggest that radiomics features extracted from 2D fetal MRI lung ROIs do not adequately represent whole lung tissue characteristics. Few features (5 of 95 [5.3%]; 10Percentile, Mean, RootMeanSquared, Median, 90Percentile) showed excellent reproducibility between the use of 2D and 3D ROIs in fetal MRI data from a single acquisition. Moreover, these features all reflected basic first order statistics but none shape or higher-order texture, effectively excluding potentially crucial information contained within the lung’s microstructure from further analysis. Another essential finding concerns the limited radiomics feature reproducibility for the use of 2D lung ROIs in repeated fetal MRI acquisitions. Here, excellent reproducibility was found in only twelve of 95 (12.6%) features. Notably, this was achieved using standardised 2D ROIs that covered the entire lung tissue except hilar structures on an axial fetal MRI slice at the level of the carina. Subjective segmentation of representative-appearing lung areas may have further increased radiomics feature variability. In contrast, excellent radiomics feature reproducibility was found in a majority of features (49 of 95, 51.6%), including a variety of shape and higher-order texture features, if 3D lung ROIs were used for both fetal MRI acquisitions.

The complex and dynamically changing microstructure of the fetal lung are unlikely to be adequately reflected by one quantitative parameter, which may explain the limited utility of previously explored lung signal intensity ratios for outcome prediction [23, 24]. Rather, a combination of images markers, i.e. ‘a radiomics signature’ focusing on different fetal lung characteristics, such as texture and shape, may be more suitable as a quantitative descriptor of lung development. Undoubtedly, lung volume remains an essential parameter to determine whether lung development is age-appropriate. By adding texture and shape features to a lung radiomics signature, the sensitivity of fetal MRI for the detection of abnormal lung development may be increased, e.g. in cases where lung volume is within normal ranges but lung microstructure is altered. As demonstrated, whole lung fetal MRI radiomics enables the extraction of a large number of highly-reproducible lung shape and texture features that may be used to develop radiomics signatures of normal and pathologic lung development in the future.

As a proof of concept, we tested whether 3D and 2D ROIs were sufficient to detect significant differences between radiomics features extracted from fetal lungs with normal or pathological development in our limited sample size. We found 11% of radiomics features were significantly different between lungs with normal compared to pathological development when using 3D ROIs. Critically, in case of the use of 2D ROIs, no significant difference in radiomics features was observed, indicating that radiomics features extracted from 3D ROIs are more sensitive to subtle, visually not perceivable changes in the fetal lung’s microstructure. Further studies are necessary to confirm these findings and identify robust and predictive 3D fetal lung radiomics features.

This study had several limitations: The number of included cases was relatively small but similar or larger compared to previous test–retest studies on radiomics feature reproducibility [13–15]. A single 1.5 T MRI scanner was used. Due to the retrospective study design, routinely performed repeated MRI acquisitions from a single examination were utilised rather than repeated fetal MRI scans. While T2-weighted images are widely used for visual assessment of the fetal lung [34], it is not known which MRI sequence is best suited for radiomics-based analysis. The radiomics feature set included in the current analysis is limited, but is compatible with the Image Biomarker Standardisation Initiative guidelines [30]. Pyradiomics has been widely used in lung imaging and beyond facilitating comparability and generalizability of results [18]. Excellent radiomics feature reproducibility was conservatively defined as ICC > 0.9, but there is a lack of evidence concerning an optimal cut-off value.

## Conclusion

In conclusion, this study demonstrates that fetal MRI radiomics features extracted from 2D ROIs do not adequately represent tissue characteristics of 3D ROIs encompassing the whole lung. In addition, they exhibit insufficient reproducibility between repeated standardised fetal MRI acquisitions. In contrast, a majority of fetal MRI radiomics features extracted from 3D whole lung segmentation masks are excellently reproducible. Therefore, highly-reproducible whole lung radiomics features represent potential image biomarkers for improved fetal MRI-based prediction of abnormal lung development and neonatal respiratory function.

## Supplementary Information

**Additional file 1. **Supplementary material.

## Data Availability

All data generated or analysed during this study are included in this published article (and its Additional file 1).

## Abbreviations

2D: Two-dimensional
3D: Three-dimensional
GLCM: Grey level co-occurrence matrix
GLDM: Grey level dependence matrix
GLRLM: Grey level run length matrix
GLSZM: Grey level size zone matrix
GW: Gestational weeks plus days
ICC: Intra-class correlation coefficient
IQR: Interquartile range
MRI: Magnetic resonance imaging
PACS: Picture archiving and communication system
ROI: Region of interest
